# Physiology-driven reversible dynamic synthetic tissue model for responsive moulage in infected wound training

**DOI:** 10.1371/journal.pone.0333565

**Published:** 2026-02-25

**Authors:** Alex T. Gong, Aleah M. DeSchmidt, Austin J. Baird, Jessica G. Gonzaga, Shi-Wen Olivia Yau, Rudolph J. Toepfer, Jessica Zhang, Conner J. Parsey, Rainer Leuschke, Jack E. Norfleet, Robert M. Sweet

**Affiliations:** 1 Department of Surgery, Division of Healthcare Simulation Science, University of Washington, Seattle, Washington, United States of America; 2 Medical Simulation Research Branch Simulation and Training Technology Center, United States of America Army DEVCOM Soldier Center, Orlando, Florida, United States of America; University of Central Florida, UNITED STATES OF AMERICA

## Abstract

Misidentification of dermatologic manifestations of systemic infection can lead to life-threatening developments including sepsis. Training medical providers to accurately identify and respond to infections using simulation could reduce these rates. Moulage of infections are static and require a manual “scene change” during simulation scenarios. A high-fidelity, automated physiology-driven dynamic infection model was developed using the Modular Healthcare Simulation and Education platform that physically changes to simulate the progression of an infected wound. The silicone model was designed using resistive wire, wax actuators, and thermochromic powder. The resistive wire heats the wax, resulting in focal edema, skin warmth, and erythema that reverses with proper recognition and treatment. Without external treatment, purulence is released, and the patient will develop septic physiology. To evaluate the physiological realism and training value of the model, a survey of physicians was conducted using a five-point Likert scale of agreement with 5 being the highest rating. When asked to rate the realism of the model, the mean response was 4.1 ± 0.7. When asked if they thought the model had value for training infection identification, the mean response was 4.3 ± 0.9. Finally, when asked if the dynamic aspect would improve the simulation, the mean response was 4.7 ± 0.6. The dynamic infection model is functional and appealing for practitioners to assist in the early detection of infection. The use of a dynamic training model could potentially be used to replicate other dermatologic manifestations of systemic disease processes and improve medical training.

## Introduction

The skin is the largest organ of the human body and functions as a barrier to the outside world. In the event of an intrusion, dermatological conditions can manifest on the skin through either the direct involvement of an infectious substance or a specific or non-specific reaction to an infection [1]. For example, the Center of Disease Control and Prevention in the United States has identified that approximately 1.7 million hospitalized patients obtain health care acquired infections (HCAIs) while being admitted for other health issues, and more than 98,000 patients die from complications due to HCAIs [2]. In addition to delayed wound healing, the inability to identify signs of a worsening or antibiotic-resistant infection can lead to the development of sepsis [3,4]. According to the World Health Organization, in 2017, sepsis was responsible for an estimated 20% of global deaths [5]. Being unable to identify early signs of a wound infection also greatly increases the cost of treatment and the likelihood that the infection will become drug resistant, increasing the cost and difficulty of treatment [4]. It is vital to train medical personnel to identify signs of a worsening infection to treat these injuries effectively.

A practice that could decrease the incidence and mortality rates of preventable HCAIs is the incorporation of simulation-based medical training, which utilizes experiential learning to improve and practice various skills. One study has found that trainings including manikin-based simulations significantly improved the students’ skills with managing mechanical ventilation [6], and that integrating physical simulations in a graduate-level medical training program significantly increased the students’ final scores and clinical performance [7]. Higher fidelity simulators have the potential to be more effective at teaching medical skills than lower fidelity models and have been shown to be more effective than traditional lecture-based teaching in certain cases [6–8]. Current wound simulators have focused on various training for medical professionals, including debridement, burn wound care, and bullet wound care [9]. There are few simulators on the market that can train medical professionals and staff to recognize and/or treat infections.

Despite the recent evidence of physical simulations benefiting the learner, there have been few advances in the ability for the educator to construct realistic wound care simulators. In general, all wound care simulators are moulage variants presented upon either a human participant or placed on a commercial mannikin [10]. Limitations of these simulators are twofold: they don’t provide feedback to the learner, and they don’t present physical properties that would be apparent in an actual patient case. These physical properties include temperature, live expulsion of fluid, and changes in coloration during a procedure. Due to these limitations, we find multiple studies that show positive learner experiences when using wound moulage during a simulation but no studies that show clinical knowledge or performance benefits [11]. Further learner benefits are observed when assessing the fidelity of the moulage being used in a simulation with 3-dimensional wound moulages reporting higher engagement than paper [12]. This type of study quantifies the need for more realistic, responsive moulage for advanced wound training to increase engagement and more physiological responsiveness to potentially impact clinical knowledge retention during a simulation.

To represent a dermatologic manifestation of a disease, a simulation technician is required to artistically render the dermatologic manifestation on a manikin or on a virtual patient prior to the scenario or to interrupt the scenario and create it as the scenario progresses. The aim of this project was to develop an automated reversible infected wound simulator that can be used to train emergency medical personnel to recognize the early signs of infection and treat it. Physiological signs prompt the search for a wound in a patient. Representative dynamic simulated tissue changes associated with infection include reversible formation of edema (swelling) and localized warmth and reddening of the skin, and the non-reversible release of purulence. By incorporating reversibility into the system, monitoring and treatment of the wound can be visualized on the model.

Reversible stimuli responsive materials are used to replicate the physiology in the wound model. Several soft actuator designs for applications in soft robots exist, utilizing fluid and wax filled thermally responsive materials [13–15]. Most involve creating pressure in parts of the system to create swelling in specific directions to achieve movement or force generation. Swelling can also be produced with the phase change of wax from a solid to a liquid with the application of heat [16]. The expansion increases pressure on the silicone and leads to swelling. Thermally responsive color-changing pigment can leverage the phase change mechanism to represent reddening of the skin while micro-openings enable the release of purulence in the form of liquified wax. The phase change required for expansion, color change, and release of purulence can be triggered electronically using nichrome wires as resistive heaters. Nichrome wires have been reliable in achieving desired temperatures in previous applications for soft actuators [13]. The electrically controlled temperature modulation of the actuation (swelling), color change and warming of the skin, and release of purulence enables easy control. To demonstrate the capabilities of the dynamic synthetic tissue concept, we built it to be compatible with the open-source Modular Healthcare Simulation and Education System (MoHSES) platform [17,18]. In doing so, it could integrate with the open-source physiology engine BioGears to drive infection progression or regression with appropriate treatment, as well as physical changes in the trainer [19]. To our knowledge, this is the first model to automate and integrate all four of these physical signs of a dermal infection into a single task trainer driven by a physiology engine.

The model utilized automated dynamic moulage to present the symptoms of an infection, with the intent of increasing the fidelity of the simulator. Much of the feedback of current simulation trainings from participants list poor tactile sensation, tissue behavior, and visual error or unrealistic appearance as the main detractors of the simulators [20–22]. The realistic appearance of simulations is enhanced by moulage, which helps participants be increasingly engaged in the simulation experience [20,22]. Higher engagement levels have been associated with higher levels of retention [22]. Simulation models of dynamically evolving infected wounds may better educate and prepare healthcare workers for early wound identification and treatment.

## Methods

### Development of the dynamic simulated tissue trainer

**Fig 1:**
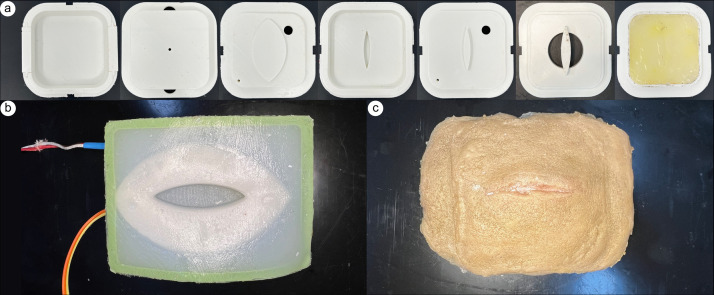
A dynamic physical model was developed to replicate the physiological changes occurring due to the progression of an infected wound. a) A series of molds were designed, and 3D printed to create each layer of the simulated tissue. The task trainer includes (b) a permanent base and c) disposable skin.

The physical trainer was developed following the Center for Research in Education and Simulation Technologies (CREST) simulation development process [23]. A series of molds (Fig 1) were designed to manufacture each layer of the simulated tissue using SolidWorks (Dassault Systèmes, Paris, France). A Prusa i3MK3 fused deposition modeling (FDM) 3D printer was used to print the molds in PLA (Polylactic Acid) (Prusa Research, Prague, Czechia). A laceration wound was modeled in clay as a relief mold and cast into the base mold using silicone rubber to represent the positive of the wound. Then, P70 silicone rubber (Silicone’s Inc, NC, USA) was prepared and degassed according to the manufacturer directions and injected into the base layer of the mold (Fig 1). 203.2 cm of 26 Gauge Nichrome (NiCr) wire was coiled into seven rows to localize heating around the wound.

After the silicone rubber was cured, the J-type thermocouple (McMaster-Carr, CA, USA) and NiCr wire were placed into the base. Hardener (Polytek, PA, USA) was mixed into Platsil Gel 25 (Polytek) at a 1:1:1 ratio and cast over the wire and thermocouple. A cavity for the wax actuator was then prepared by using the lid of the mold (lid one).

Hardener (Polytek) was mixed into Platsil Gel 25 (Polytek) at a 1:1:1 ratio and injected into the mold. Once cured, lid one was removed and 5.0 g of paraffin wax (McMaster-Carr) was melted and poured into the cavity. After the wax was resolidified, the second lid was placed on the mold and Platsil Gel 0020 (Polytek) was prepared per manufacturer directions and injected into the mold. Once fully cured, the skin was demolded and a tacky layer of silicone rubber was prepared to adhere the replaceable skin layer to the base. LV Deadener (Polytek) was mixed into Ecoflex Gel (Smooth-on, PA, USA) and poured onto the top surface of the base completing the permanent base (Fig 1b).

The disposable skin layer was then created (Fig 1c). Platsil Gel 0020 was prepared per manufacturer’s directions and cast into the skin mold. 1.0 g of paraffin wax was melted, colored with yellow wax dye and TiO2, and cast into the cavity. Once solidified, white to red thermochromic powder (SolarColorDust, FL, USA), Silc Pig Skin and Brown pigment (Smooth-on, PA, USA), and rayon flocking (Flocking Unlimited, USA) were added to Platsil Gel 0020 prior to injecting into the mold to create the external skin surface. Once fully cured, the skin was demolded and adhered to the base with a tacky layer of silicone rubber. LV Deadener (Polytek) was mixed into Ecoflex Gel (Smooth-on) and poured onto the bottom surface of the skin layer similar to the process described in the previous section.

### Physiology driven wound progression of the infected wound model

**Fig 2:**
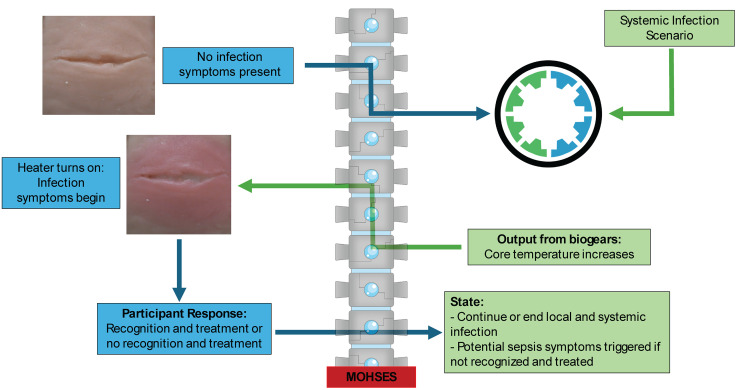
Graphic describing the dynamic infection model with the MoHSES platform and BioGears engine.

The physical system is integrated into BioGears to model the progression of a bacterial infection and systemic inflammation using the MoHSES platform [19,24,25], (Fig 2). This platform provides a bridge between the physiological model and the system using a serial bridge and an Arduino microcontroller.

The infection model of the patient begins with an initial infected state, declared to be either mild, moderate, or severe and an initial minimum inhibitory concentration, classifying the bacterial interaction with an antibiotic agent. The model assumes a Streptococcus pneumoniae inoculum that initially colonizes the lungs, unless initially specified by the user. Configuration parameters for the model include location of the pathogen before systemic release into the bloodstream. Other parameters include general patient parameters and initial bacterial count, which we classify into either “mild,” “moderate,” or “severe.”

**Fig 3:**
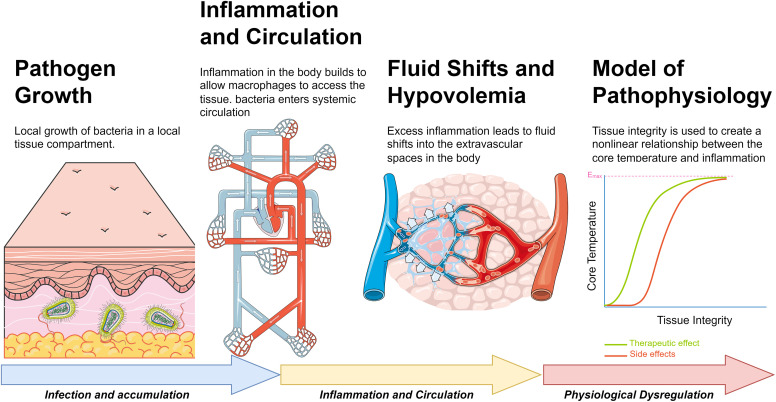
Overview figure denoting the model progression of sepsis. We can leverage the accumulation of these interacting models to extract a core temperature throughout the simulation that is a function of these prior effects. For more severe bacterial infection, the tissue damage leads to more severe thermoregulatory changes.

For this specific study, the simulated patient began with a “moderate” infection, which constitutes an initial pathogen amount (count) of 50 million. A “mild” infection indicates 10 million initial bacterial count and a “severe” infection equals a 250 million initial bacterial count. The patient was then simulated for 36 hours while the infection influenced the patient inflammatory and immune responses. During this simulated time, the patient was given food and water. Inflammation creates a degradation of the tissue microvasculature, allowing macrophages to interact with the bacteria (Fig 3). This breakdown of the capillary lumen provides access for the bacteria to enter systemic circulation. The inflammatory cascade progresses due to this systemic circulation, creating more fluid shifts into the tissues of the body, causing hypovolemia. The microcontroller extracts the core temperature of the patient during progression of the bacterial infection to control when heating of the wound model should occur emulating a fever. Once the core temperature rises at infected levels, the other physical signs of infection (edema, erythema and purulence) are heat-activated on the physical model. In the event of an intervention (e.g., correct application of antibiotics), the microcontroller can reverse the effects of an edema and erythema through disabling the heating element, ultimately leading to a reduction in core temperature of the patient. As this specific study was only evaluating the visual and tactile presentations of the infection, the simulated patient was not provided reversal agents such as fluids or antibiotics, and the simulation was finished once the evaluation study was completed (approximately 2–5 minutes).

The temperature of the patient is modified using an Emax function that relates the core temperature and the tissue integrity of the microvasculature.


$$E=\frac{E_{max}(\text{1}-TI)}{E_{\text{5}\text{0}}^{\gamma }+(\text{1}-TI)}$$

Here E denotes the pathophysiological “effect,” with a half and maximal parameterization, TI denotes the tissue integrity of the capillary microcirculation. For the purposes of our physical model, E denotes the core temperature of the patient. The tissue integrity is a culmination of the inflammatory cascade that results from systemic pathogen growth and is a function of interleukin 6 and nitric oxide.


$$\frac{dTI}{dt}=k_{D}(\text{1}-TI)\left(TI-{TI}_{min}\right)-k_{D}\left(TI-{TI}_{min}\right)\left(\frac{{IL\text{6}}^{\text{6}}}{{IL\text{6}}^{\text{6}}+x_{D\text{6}}}\right)\left(\frac{\text{1}}{x_{DNO}^{\text{2}}+{NO}^{\text{2}}}\right)$$

Here kd, xD6 and xDNO are parameters IL6 is interleukin 6 and NO denotes nitric oxide. We omit full details of the model of infection and inflammation used for this project for brevity, a complete discussion can be found for the reader [25].

### Pilot Study: Evaluation of the physiological realism and educational benefit of the trainer

A pilot study using a sample of convenience was conducted to evaluate the physiological realism and training value of the model. A narrated timelapse video of the benchtop model was created (Supplemental information 1) to demonstrate the progression of a wound infection. Afterwards, the participants were asked to complete a survey consisting of a series of questions to be scored on a five-point Likert scale of agreement with 5 being the highest rating. Finally, the scores were averaged across specialty to determine how the model scored for each question. The anonymous opt-in survey was distributed through email among surgery groups at the University of Washington Medical Center between February 22^nd^, 2022 – March 1^st^, 2022. The survey questions consisted of the following:

1. On a scale of 1–5, please rate how realistically the model portrays an infected laceration wound?
2. On a scale of 1–5, how much do you agree with the statement that this model has value for training in the recognition of the signs of infection?
3. On a scale of 1–5, please rate how much you think the dynamic aspect of the model affects the learning experience of the simulation when compared to a static infected wound model that does not change?

## Results

### Physiology driven wound progression of the infected wound model

**Fig 4:**
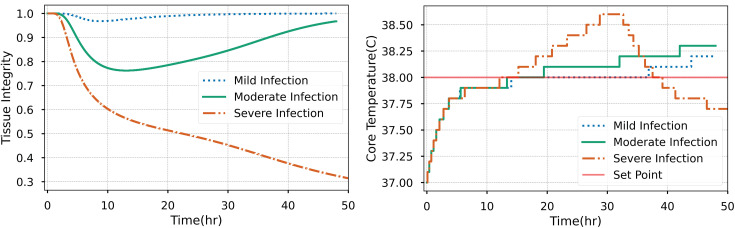
The simulated patient’s physiological response to varying levels of initial bacterial populations in the tissue. The core temperature and tissue integrity are reported for each. The set point is the threshold by which the heating in the physical model is triggered.

Fig 4 displays the results of a patient exposed to mild moderate and severe bacterial colonization in the tissue, as an input into the dynamic infection model. For the mild and moderate cases (10 million and 50 million initial pathogen count), the body can clear the infection naturally due to the macrophage activity. The severe case (250 million initial pathogen count) progresses to sepsis and eventually patient death. The core temperature increases much more rapidly for this patient, especially in the early stages of infection. The set point resulting in a physical manifestation of wound infection is shown for each patient at around 14 hours of simulation time. Currently, the physical device doesn’t consider an adjustment due to the magnitude of core temperature increase but could be considered in future research.

In the model, the core temperature is a function of tissue integrity, and we can see that for the three patient cases the severe case leads to an uncompensated fluid shift into the tissue due to capillary permeability changes. These changes are due to accumulation of inflammatory markers in the body, detailed in equation 2. This bifurcation in patient response is due to the initial conditions in the model as a “moderate” and “mild” infection denotes a reduction in overall bacterial populations in the tissue.

**Fig 5:**
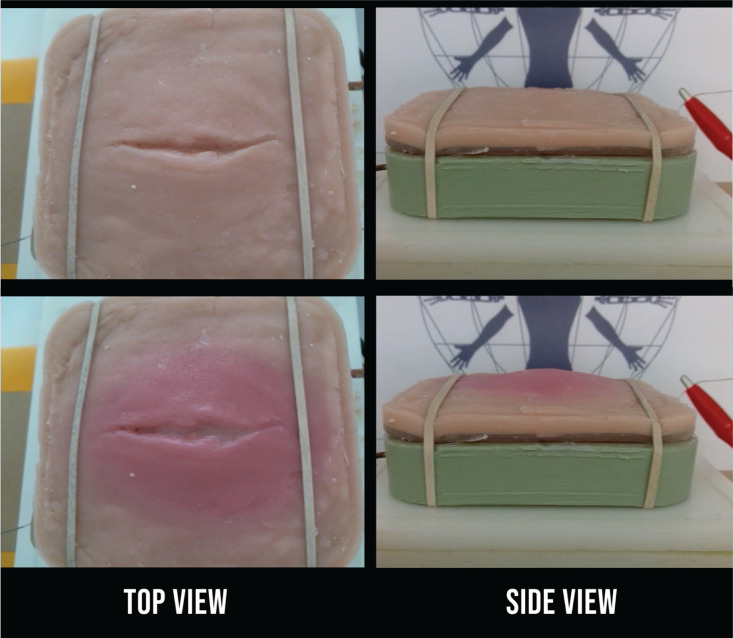
As the infection progresses from a) initial wound to b) infected, the simulated tissue undergoes physical changes resulting in the surface temperature increasing to feverish levels, reddening of the skin around the wound, localized formation of an edema, and ultimately the release of purulence.

When the physical model is triggered by the physiology engine to begin at 38˚C, the set point temperature is reached for the patient model, the dynamic changes begin (Fig 5). Internal temperature linearly increases to 70˚C in 40 minutes where it is limited by the relay. During this time, the reddening of the skin and formation of an edema occurs at the 10-minute mark, when it would be 45° C internally. The erythema began to appear at the 25 minute-mark, at 70° C internally, and the purulence started soon after. The surface temperature of the skin plateaus at 40˚C, reflecting feverish skin.

### Pilot Study: Evaluation of the physiological realism and educational benefit of the trainer

**Fig 6:**
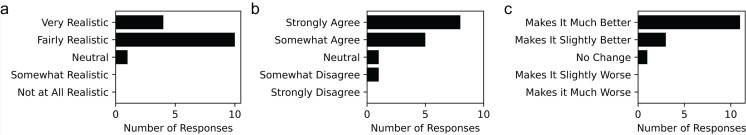
a) Survey responses to question one: On a scale of 1 to 5 with 1 being “Not at All Realistic” and 5 being “Very Realistic”, please rate how realistically the model portrays an infected laceration wound? b) Survey responses to question two: On a scale of 1 to 5, with 1 being “Strongly Disagree” and 5 being “Strongly Agree”, how much do you agree with the statement that this model has value for training in the recognition of the signs of infection? (c) Survey responses to question three: On a scale of 1 to 5, with 1 being “Makes it Much Worse” and 5 being “Makes It Much Better”, please rate how much you think the dynamic aspect of the model affects the learning experience of the simulation when compared to a static infected wound model that does not change.

Fifteen surgeons responded to the survey, Table 1. The survey results are shown in Fig 6.

**Table 1:** Participant’s surgical specialty.

Specialty	Number of Participants
Plastic Surgery	6
General Surgery	5
Urology	2
Unreported	2

When asked to rate how realistically the model portrayed an infected wound on a scale of 1–5, the mean response with a scale of one being not at all realistic and five being very realistic was 4.1 ± 0.7. When asked if the participants agreed that this model had value for training in the recognition of the signs of infection, the mean response of a scale of one being strongly disagree and five being strongly agree was 4.3 ± 0.9. The final question was how the respondents thought the dynamic aspect of the model affected the learning experience and the mean response on a scale of one being makes it much worse and a rating of five being that it makes it much better was 4.7 ± 0.6.

## Discussion

This paper emulated several dermatologic manifestations/characteristics of an infected wound. The edema was achieved using the encapsulated wax and produced consistent, uniform moderate swelling once the target internal temperature was achieved. Purulence was produced by a colored wax mixture and was accurate in appearance, (Fig 5). The erythema was also consistent and realistic in appearance once the target internal temperature was achieved. Finally, the heating element worked to evenly heat the model and trigger all the other elements and warm the surface to between 37–40° C to simulate skin that is warm to the touch. This was successfully demonstrated in the standalone benchtop model. By utilizing a nearly fully reversible approach to physiology replication with BioGears, we can modify the effect of treatment (application of topical antibiotics) to match physical responses (decrease in swelling, surface temperature, and redness) in the physical simulation.

### Physiological model use and possible extensions

Although modeled as a specific type of bacterium, the complete sepsis model implemented in BioGears consists of various generalized physiological responses that are universally deployed to fight infection. Due to this generalization, there are means for extending this model to cover various other pathogen types that might behave, transport, or infect in different ways. Parameterization of the model can be extended and generalized to be encompassed by an external configuration file (JSON equivalent) to include parameters that would be associated with various other types of bacteria, such as: S. aureus, P. aeruginosa, or Aeromonas hydrophila. These types of bacterium would alter the initial configuration of the pathogen growth, and interaction terms to be specific to these model organisms. Additional parameters may need to either extend the existing inflammatory cascade, tissue barrier interactions or macrophage interactions as necessary given experimental knowledge. Once these steps are completed this model could be leveraged to teach a variety of different infection treatments due to different bacterium and would be a generalized resource rather than a specific model for this implementation. The open-source nature of the code base provides resources and tools for other developers to extend the model as desired.

### Comparisons to existing dynamic wound research

The most related work to the model discussed in this paper was performed under an effort from DEVCOM Soldier Center called Dynamic Wounds for Training. The Dynamic Wounds effort targeted five similar material response areas: color change, edema, temperature change, liquid secretions, and olfactory capabilities. In collaboration with SIMETRI and the University of Alabama, DoD Contract #W912CG-20-C-0033, the team sought to use a novel ionic polyamide (i-PA) formulation to create a set of dynamic wounds capable of responding to proper or improper care over time. Iterative research results included pustule wounds that could swell and secrete fluids, reversible and non-reversible methods for swelling, reversible temperature change, and reversible color change. The final prototype was a laceration wound capable of swelling, temperature change, and color change that responded based on the level of care provided and some external input from an instructor controller. The i-PA materials proved successful for one-way swelling, but a mechanical component was required to get fully reversible edema in the final prototype. Temperature change was controlled through a remote app and was achieved with a similar heating coil arrangement under the surface of the wound. While the Dynamic Wounds could respond to treatment being provided, the responses were not driven by a physiology engine such as BioGears. This Dynamic Wounds effort also researched ways of 3D bioprinting the i-PA wounds and their molds to achieve higher customization of material properties and aid in manufacturability.

Overall, there is not an extensive body of research, nor commercially available products, related to treatment responsive physical wound simulations. Industry standard full body human patient simulators still have static wound patterns that might be modular or swappable, but do not react physically to the treatment being provided. There have been a few research efforts exploring wound overlays and dynamic moulage, but these were not self-compensating (requiring some form of instructor input or external controller) or driven by a physiology engine. The vast majority of literature on dynamic tissue simulators are mechanical or computer modeling. Park et. al. developed a benchtop biorobotic mitral valve simulator to act as a testbed for testing interventions related to mitral valve repair [26]. Tang et. al. developed a finite element model that incorporated physiologic factors to better simulate deformation of live patients’ brain and liver [27]. Therefore, there exists a significant gap in literature to address dynamic wound moulage.

### Application and viability of the infection model in medical education

One possible training application for the described work is in the education of catheter related bloodstream infection management. Within a systemic review of 20 surgical simulation studies of Catheter-Related Bloodstream Infections, surgical simulation education was associated with improved learner outcomes in knowledge, confidence, and performance on the simulators and improved patient outcomes including fewer needle passes and pneumothorax needle insertions [28]. However, across the studies, there was not an association between the utilization of the available surgical simulators and reduction in risk of arterial puncture or catheter-related infections [28]. In future studies, our wound simulator can be utilized to train the visual diagnosis of infected tissue. Catheter-Related Bloodstream Infection is characterized by focal warmth/erythema, which is simulated in our model, providing a visually accurate physical infection model for this training application [29]. There is a need to determine if the realism of this model’s appearance increases fidelity of the simulation, which may increase participant engagement and knowledge retention.

Other applications of this platform include other simulated training for infection, such as sepsis related education. Recent studies revolve around team training in the clinical care of the patient and have very little physical interactions with a manikin [30]. To supplement these studies, during the stages of sepsis simulation training, our model could be utilized as the morphing aspect of the moulage to allow the learner to interact with a dynamic infection that responds to various treatments that the learner provides. Educational encounters may focus on dissipation after infection identification and administration of correct treatments, or for dissipation over time in response to mismanagement of care. In many treatment scenarios for sepsis, the dynamic nature of our model may increase the fidelity and feedback time of the simulation experiences for the teams of medical providers.

In addition to training recognition and treatment of the patient, this model may also train medical professionals and even patients to self-monitor their wounds, with the intent to lower the patient intake time in the EDs and reduce preventable adverse health outcomes due to longer wait times for treatment. Data simulation of the impacts of increased patient infection rates on overall patient intake and flow within the Emergency Departments (EDs) in hospitals found that the increased patient infection rates would increase length of stay and crowding within emergency departments as a whole [31]. Increased wait times for all patients would increase adverse health outcomes and healthcare costs to the patients and clinics. In addition, patient flow and patient intake were adversely impacted by the COVID-19 pandemic, creating a need to implement preventative measures to reduce collateral impact of patient care from pandemics [32]. As we navigate later stages of the COVID-19 pandemic, it is vital to consider how infection and sepsis can lead to increased adverse healthcare outcomes of all ED patients. For example, a study found that rapid care of sepsis through antibiotics, and administration of intravenous fluids were associated with lower risk-adjusted in-hospital mortality [33]. The implementation of the infection simulator model could potentially be used in the future as a classification tool to provide period training to healthcare workers, potentially reducing ED time.

Although HAI prevention and control programs are considered a cornerstone of all patient safety, infection prevention and intervention education are primarily conducted through classroom-based teaching internationally, without active learning strategies. The content of infection prevention and intervention education must consider the prior experience and knowledge among the diversity of healthcare specialties. The infection prevention and intervention education also primarily focuses on hand hygiene, PPE protocols, cleaning, and disinfection management [34]. These are valuable skills and training, but these do not capture the identification of infections in patients themselves. In studies related to the identification of sepsis, didactic presentations, video vignettes, pre- and post- knowledge tests, and high-fidelity medical simulation scenarios are common training tools. Studies show that post-knowledge test scores increased with the implementation of the educational program centered around sepsis [35]. Also, most educational programs were determined to be effective in immediate knowledge retention, although the utilization of active learning strategies including simulation and game-based learning generally improved understanding of sepsis more so than didactic teaching [36]. Active learning provides better retention in studies of sepsis education, and this platform provides a valuable tool to be used in an active learning environment. Evaluations of this platform have shown, on average amongst surgeons who completed a survey, a positive view of the model and agreement that it realistically portrayed an infection and could be useful in training students to recognize the signs of infection. Also, most surgeons thought the model’s dynamic aspect would greatly improve the learning experience during training. This feedback is promising and indicates that this model is realistic and could be used effectively in a training setting. Further studies comparing it to static models and textbooks currently in use could be beneficial to further support these points. The high-fidelity surgical simulator we have developed has the potential to be used in comprehensive educational programs for medical professionals to improve infection identification for sepsis and other medical conditions.

### Applications of the dynamic model in simulation

This technology establishes a proof of concept that can be applied to other simulation scenarios. Edema and erythema are two elements that are present in several medical procedures and conditions. Many dermatological conditions, including those caused by inflammatory conditions and infectious disease, have similar signs and symptoms. The color of the erythema can be easily changed by integrating other thermochromic powder options. A surgical procedure simulation could use the swelling and color change moulage to signal if a trainee has made a mistake that has irritated surrounding tissue to provide real time feedback on their technique. The model could also be used for simulating post-operative care of a surgical wound with the integration of sutures. Although the model skin color presented in this paper was designed to show the technology distinctly, this is hardly the case in the real world. It is important to have multiple models with a variety of skin tones to integrate cultural humility into the model. As some of the signs of infection included in the model may look different on individuals with different skin tones, it is important that medics are prepared to treat all patients to mitigate structural racism within medical education.

A key aspect of the dynamic synthetic tissue model is the reversibility of the physiological changes in the physical model. Aside from the purulence, once the heating source is turned off and cooled, each of the components is fully reversible. This feature enables real time response of interventions during simulation. It also enables the models use in numerous simulations before being replaced. The purulence can be easily refilled using melted wax. This capability is both ideal economically and environmentally conscious with the cost per model and waste being reduced.

This model can stand alone as a task trainer specifically for the identification and treatment of infected wounds, or it can be integrated as part of a larger manikin simulation where it can assist in teaching a variety of skills including wound management, infection identification and treatment in addition to overall patient care. Additionally, the modular nature of the concept enables more unique sources of infection – particularly useful in smaller wounds hidden amongst multiple other large injuries. It can also be expanded to include olfactory and audio cues particularly useful in military or large casualty events. In the case of this paper, the activation of the infection process can be triggered in response to information from a physiology engine like BioGears; this could work the other way around with the infection model providing information to BioGears to further activate other steps of the simulation. This may include antibiotic administration and wound packing that could directly influence the physiology model. In doing so, objective performance metrics (time to identification, correct treatment applied, etc.) by learners can be tracked for after-action review. This technology has the potential to elevate moulage used in simulations to provide a more accurate and engaging experience for medical trainees that will further contribute to the development of their skills.

## Limitations

As the pilot study received 15 responses from solely surgeons, the small sample size and lack of diversity of medical professions were limitations. In the future, we hope to survey a wider range of medical personnel, including all medical professions who could interact with the patient, including nurses, nurse practitioners, and physician assistants [37]. Self-recognition for patients and families could also be an interesting target population. In our pilot study, we also only had access to a surgical department; however, input from varying levels of patient interaction in specialties that have higher prevalence of patients with infections or patients at risk for infection would allow for comprehensive feedback regarding the usefulness of the simulator.

Future iterations of the wound model would ideally all be 3D printable, which would reduce the time and complexity of manufacturing. As more institutions adopt 3D printing into their curriculum, adoption of on-site molding and printing for training will be made available. In a study that utilized 3D printing for the creation of a mastoidectomy surgical trainer, the model was cost-effective, feasible, and enables reproduction of the model at other institutions [38]. This capability will eventually broaden manufacturing of the model to be used at various training sites and institutions. Specifically, the coloration of the device could be made more realistic with different amounts of coloring material able to be programmed in for various locations on the model, making it less uniform. Additionally, it enables the shape of the wound and overall model to be more geometrically complex and continuously altered and updated without significantly adding to production costs.

## Conclusions

This study demonstrated the ability to integrate reversible stimuli-responsive materials, driven by a physiology engine, to replicate the physical changes in an infected wound. Future work will include applying the concepts of the wound model to simulate different dermatological and critical care applications for skills training, as well as assess long-term training retention of these skills.

## Supporting information

S1 Video Survey video circulated for the pilot study. (MP4)
